# Post-infarction left ventricular pseudoaneurysm with septal rupture: a case report

**DOI:** 10.11604/pamj.2025.52.160.50101

**Published:** 2025-12-16

**Authors:** Bibissara Yerekesh, Zhannat Nurumova, Anel Assylbekova, Rustem Tuleutayev, Dmitriy Gebel, Leila Sani, Saltanat Altybayeva, Alima Turemuratova

**Affiliations:** 1 Department of Radiology and Cardiac Surgery, Research Institute of Cardiology and Internal Diseases, Almaty, Kazakhstan; 2 Department of Radiology, Almaty City Emergency Hospital, Almaty, Kazakhstan

**Keywords:** Ventricular septal rupture, pseudoaneurysm, cardiac surgery, coronary artery bypass, case report

## Abstract

Despite advances in reperfusion therapy, acute myocardial infarction may still lead to rare, life-threatening mechanical complications. We describe the case of a 58-year-old man who developed progressive heart failure two months after an untreated anterior myocardial infarction. Multimodality imaging demonstrated a large left ventricular apical pseudoaneurysm with post-infarction ventricular septal rupture into the right ventricle, resulting in a significant left-to-right shunt. The patient underwent successful aneurysmectomy, closure of the septal defect, and coronary artery bypass grafting. This report highlights a rare combination of mechanical complications following delayed reperfusion and emphasizes the essential role of imaging in diagnosis and surgical planning. Given its rarity and complexity, this case contributes to the understanding of structural sequelae of myocardial infarction.

## Introduction

Despite significant progress in reperfusion therapies, acute myocardial infarction (AMI) can still result in severe, potentially fatal complications. Among these, post-infarction ventricular septal defect (PI-VSD) remains one of the most critical, and its combination with a left ventricular (LV) apical pseudoaneurysm rupturing into the right ventricle is exceedingly rare [1-3]. Historically, PI-VSD occurred in 1-2% of AMIs, but thrombolytic therapy has reduced the incidence to ~0.2%. However, in-hospital mortality remains high: ~45% with surgery, up to 90% without [4]. Risk factors include cardiogenic shock, right ventricle dysfunction, and inferior infarcts [4]. Surgical repair is the mainstay of treatment, but determining the optimal timing and addressing the need for concurrent revascularization can be challenging. We report a rare case of post-infarction septal rupture with apical pseudoaneurysm and left-to-right shunting, managed successfully with surgical repair and coronary bypass.

## Patient and observation

**Patient information:** a 58-year-old man was admitted to our hospital 2 months after an acute myocardial infarction (AMI). He experienced retrosternal chest pain on October 10, 2026, but delayed seeking medical attention for 14 days and was initially hospitalized at a regional center. Previous coronary angiography on October 26, 2026 showed a right-dominant circulation with 85-90% proximal LAD stenosis and acute mid-LAD occlusion. Multiple balloon inflations were attempted, but no-reflow/slow-reflow occurred due to a transmural anterior LV infarction, and stenting was deferred because of high thrombosis risk. The patient´s history included hypertension, type 2 diabetes mellitus, ischemic stroke in 2022, and varicose veins of the lower limbs. At presentation, he reported progressive dyspnea at rest, orthopnea, lower-limb edema, and ascites. He denied smoking and used alcohol only occasionally.

**Timeline of current episode:** on October 10, 2024, the patient experienced retrosternal chest pain but did not seek immediate medical care. On October 24, 2024, he was admitted to a regional hospital, where he was diagnosed with an acute anterior myocardial infarction. Coronary angiography performed on October 26, 2024, revealed 85-90% stenosis of the proximal left anterior descending artery (LAD) and an acute occlusion in the mid-LAD segment. Balloon angioplasty was attempted; however, a no-reflow phenomenon occurred, and stent implantation was not performed due to unfavorable conditions. On December 19, 2024, the patient was admitted to our facility with decompensated chronic heart failure, presenting with dyspnea at rest, orthopnea, peripheral edema, and ascites. Comprehensive evaluation included electrocardiography, laboratory tests, echocardiography, and contrast-enhanced computed tomography angiography. On December 23, 2024, the patient underwent open-heart surgery, which included closure of the post-infarction ventricular septal defect (PI-VSD), the Dor procedure, and left internal mammary artery (LIMA) to LAD bypass grafting. The postoperative course was uneventful, with significant symptomatic improvement, and the patient was subsequently transferred to a cardiac rehabilitation program.

**Clinical findings:** at presentation, the patient was in moderate-to-severe condition due to decompensated chronic heart failure. He was orthopneic but conscious and hemodynamically stable (BP 120/70 mmHg, temperature 36.4 °C). Peripheral edema and ascites were evident. No anginal pain was reported.

**Figure 1:**
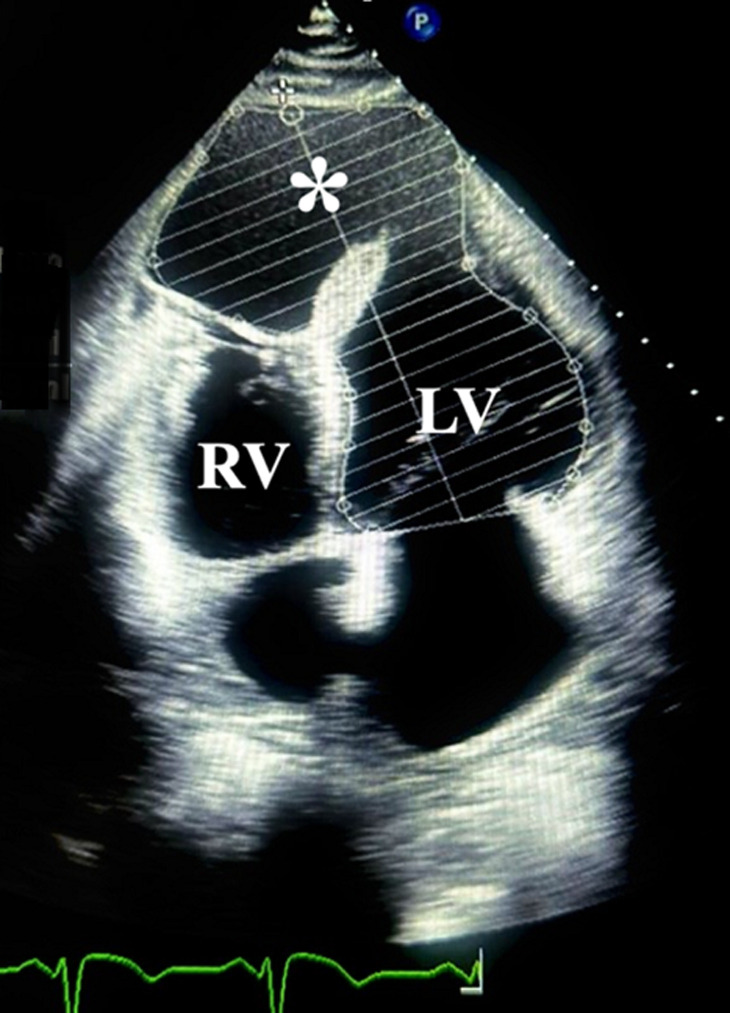
echocardiogram, 4-chamber view showing pseudoaneurysm (*) communicating with the left ventricle (LV), right ventricle (RV)

**Figure 2:**
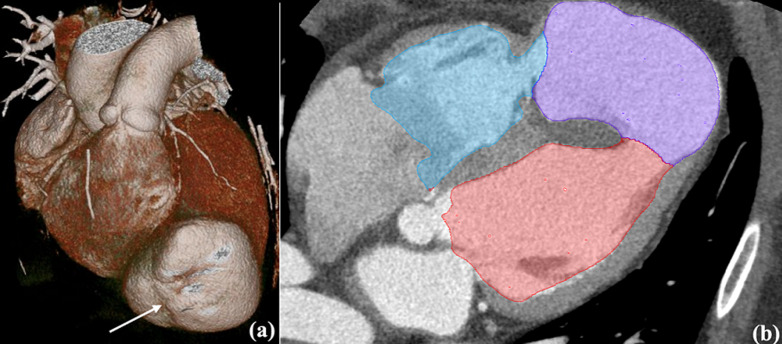
cardiac computed tomography angiography (CCTA); A) 3D reconstruction, where pseudoaneurism is shown with an arrow, and B) four-chamber long-axis view - showing apical pseudoaneurysm (violet) connected to the left ventricle cavity (red) and to the right ventricle (blue); dilated chambers and serous pericardial effusion are also seen

**Diagnostic assessment:** transthoracic echocardiography revealed a large apical pseudoaneurysm (6.6 × 8.1 cm) and a 1.2 cm post-infarction ventricular septal defect (PI-VSD) with predominantly left-to-right shunting. Left ventricular ejection fraction (LVEF) was 26%, with pulmonary hypertension (rSPAP 76 mmHg) (Figure 1). Cardiac computed tomography angiography (CCTA) confirmed a 7.1 × 8.1 cm pseudoaneurysm communicating with both ventricles, chamber dilation, RV hypertrophy, and moderate pericardial effusion (Figure 2). Follow-up coronary angiography and ventriculography showed persistent proximal and mid-LAD stenosis (85-90%), 40% mid-distal Cx stenosis (TIMI III), and unobstructed RCA (TIMI III). Left ventriculography revealed a pseudoaneurysm measuring 92 × 73 × 81 mm with hypokinesia of the anterior, anteroseptal, and lateral walls; EDV 221 mL, ESV 79 mL, EF 65%. Electrocardiography demonstrated sinus rhythm, HR 83 bpm, pathological Q waves in V1-V6, flattened T waves in I, II, III, aVL, aVF, and persistent ST-segment elevation in V2-V5, consistent with prior anterior MI. High-sensitivity troponin T was 25.3 ng/mL. Laboratory assessment revealed elevated cardiac troponin, systemic inflammation (increased CRP levels), mild hepatic enzyme elevation, moderate renal impairment (CKD C3a), and subclinical hyperthyroxinemia.

**Diagnosis:** multivessel coronary artery disease with angina pectoris (NYHA III). Transmural anterior wall myocardial infarction with ST-segment elevation (October 24, 2024), complicated by left ventricular apical aneurysm and post-infarction ventricular septal defect. Severe pulmonary hypertension and chronic heart failure with reduced ejection fraction (26%, NYHA III, ACC/AHA stage C).

**Figure 3:**
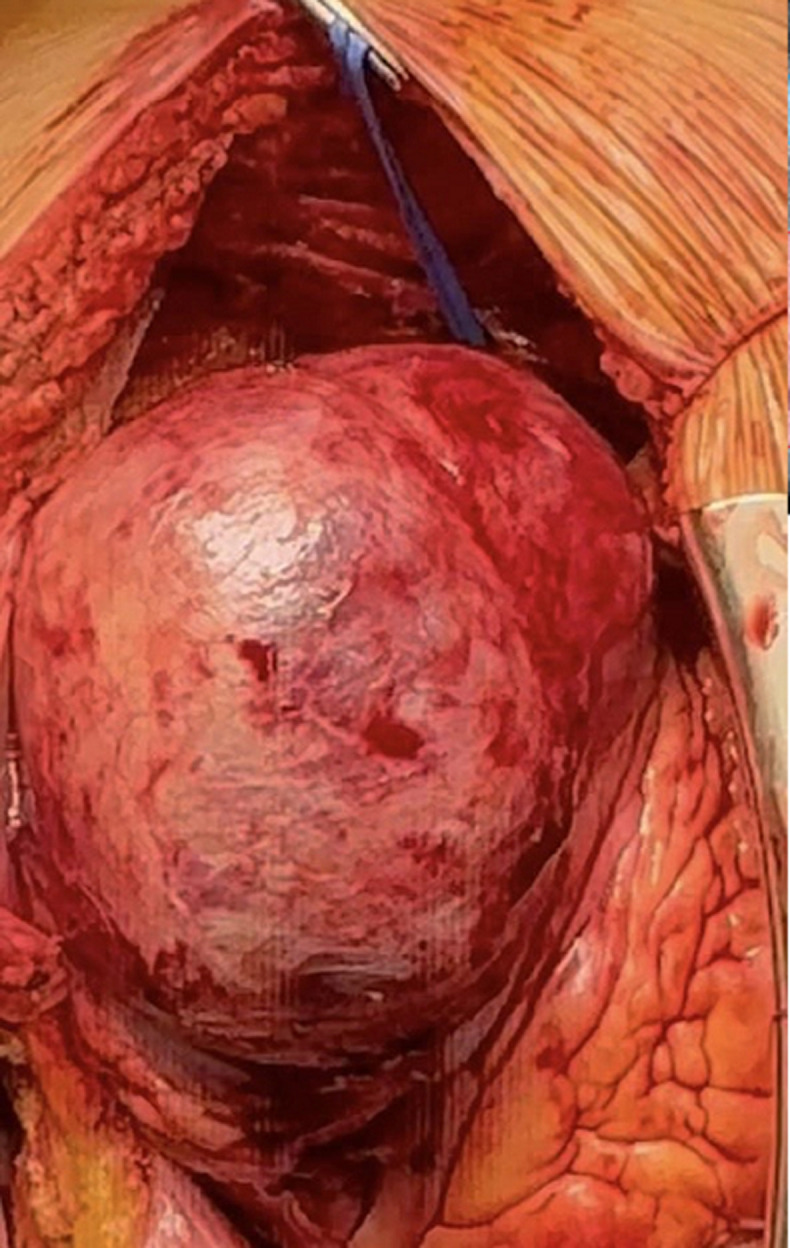
intraoperative photograph demonstrating the performed thoracotomy; pseudoaneurysm measuring 7.0 x 5.5 cm is visible in the antero-apical region of the left ventricle

**Therapeutic interventions:** on December 23, 2024, the patient underwent open-heart surgery. A large LV apical pseudoaneurysm and a post-infarction VSD were identified (Figure 3). The septal defect was closed with pledgeted sutures, followed by left ventricular remodeling (Dor procedure) and LIMA-to-LAD bypass grafting. Postoperative echocardiography confirmed successful shunt closure and graft patency.

**Figure 4:**
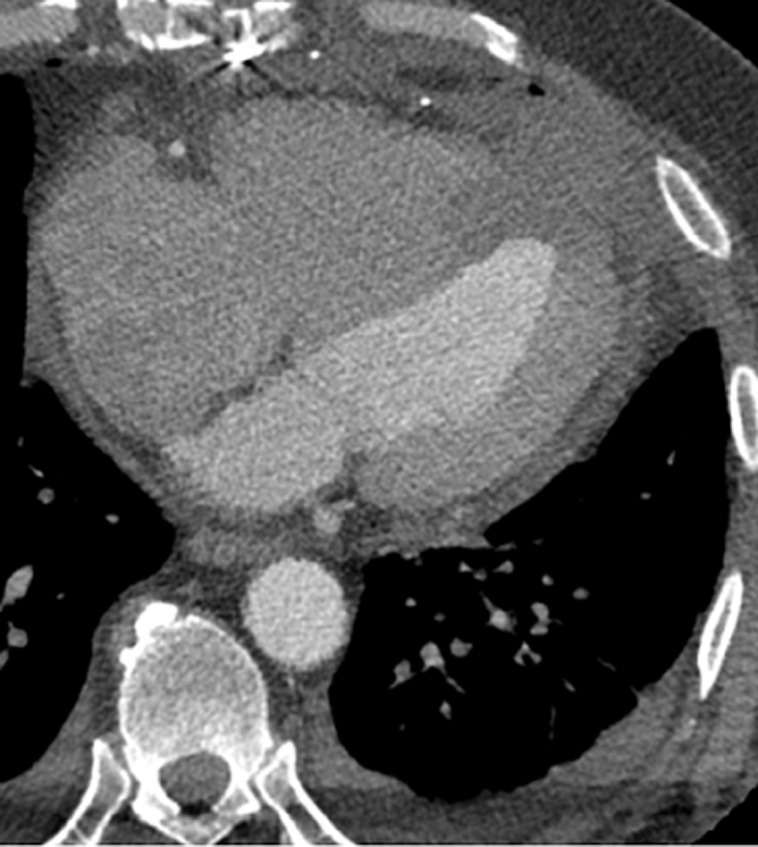
postsurgical computerized tomography after left ventricular remodeling with xenopericardial patch (Dor Procedure); pericardial and bilateral pleural effusion present

**Follow-up and outcome of interventions:** the patient was weaned from cardiopulmonary bypass without complications. Recovery was uneventful, with gradual improvement in symptoms, mobility, appetite, and resolution of dyspnea at rest. He was transferred to a cardiac rehabilitation unit, and repeated cardiac CT angiography confirmed successful repair (Figure 4).

**Patient perspective:** the patient expressed relief and satisfaction after the surgical intervention, noting a marked improvement in breathing, mobility, and overall well-being. He appreciated the thorough explanation of his condition and the steps of the surgical procedure, and reported feeling reassured by the multidisciplinary care he received during hospitalization and rehabilitation.

**Informed consent:** written informed consent was obtained from the patient prior to any interventions. The patient was fully informed about the risks, benefits, and potential outcomes of the treatment.

## Discussion

Mechanical complications following acute myocardial infarction (AMI) encompass myocardial wall rupture, papillary muscle rupture, and the development of ventricular aneurysms [5]. Their clinical presentation varies from mild dyspnea and hypotension to profound cardiogenic shock, depending on the extent and rapidity of myocardial damage. Although reperfusion therapy has significantly reduced their incidence (<0.1%), mortality remains high [6]. The incidence of post-infarction ventricular septal defect has markedly declined since the introduction of reperfusion therapy, currently estimated at approximately 0.2% of acute myocardial infarctions [1-3]. However, our case illustrates that despite this decline, PI-VSD remains a devastating complication with high mortality. Early diagnosis and hemodynamic stabilization are therefore essential. Echocardiography serves as an important tool for evaluating ventricular performance and detecting left-to-right shunting, while contrast-enhanced cardiac CT angiography enables comprehensive anatomical visualization to guide diagnosis and operative strategy. Surgical repair remains the standard of care for post-infarction ventricular septal defects (PI-VSD), with percutaneous closure serving as an alternative or bridge to surgery [6]. This case highlights a rare and complex mechanical complication: a large left ventricular apical pseudoaneurysm combined with PI-VSD causing significant left-to-right shunting. The complication resulted from a massive transmural anterior myocardial infarction with delayed reperfusion therapy [7]. Rupture of the apical myocardial wall was contained by adhesions to the pericardium, forming the pseudoaneurysm and creating an abnormal communication between the left and right ventricles. This worsened hemodynamics and caused pulmonary hypertension, necessitating urgent surgical intervention. The combined surgical approach of aneurysmectomy, PI-VSD closure, and coronary artery bypass grafting successfully restored ventricular geometry and function.

## Conclusion

This case illustrates a rare combination of post-infarction mechanical complications-ventricular septal rupture with left-to-right shunting and a left ventricular apical pseudoaneurysm-resulting from delayed reperfusion after a massive anterior myocardial infarction. It emphasizes the role of timely surgical intervention and the importance of advanced imaging for accurate diagnosis in achieving successful outcomes.
